# *Exophiala lecanii-corni* keratitis presenting as a serpiginous pigmented superficial lesion: a case report

**DOI:** 10.1097/MD.0000000000022121

**Published:** 2020-09-04

**Authors:** Tomoko Miyakubo, Daisuke Todokoro, Yoshiyuki Satake, Koichi Makimura, Sumiko Miyakubo, Hideo Akiyama

**Affiliations:** a Department of Ophthalmology, Gunma University, 3-39-15 Showa-machi, Maebashi, Gunma; b Tokyo Dental College Ichikawa General Hospital, 5-11-13 Sugano, Ichikawa, Chiba; c Miyakubo Eye Clinic, 2-3-15 Aramaki-machi, Maebashi, Gunma; d Medical Mycology, Graduate School of Medicine, Teikyo University, Itabashi-ku, Tokyo, Japan.

**Keywords:** black mold, descemet stripping automated endothelial keratoplasty, *Exophiala lecanii-corni*, fungal keratitis, voriconazole

## Abstract

**Rationale:**

Patients with bullous keratopathy (BK) treated by Descemet stripping automated endothelial keratoplasty (DSAEK) have a compromised cornea, due to the administration of topical steroid, postsurgical use of contact lenses, and impaired barrier function of the corneal epithelium by BK. We report a case of *Exophiala lecanii-corni* (*E lecanii-corni)* keratitis presenting as a serpiginous pigmented superficial lesion after DSAEK.

**Patient concerns:**

An 81-year-old woman who had undergone cataract surgeries, suffered from decreased vision in the left eye. She was diagnosed BK and she underwent DSAEK. Two months after DSAEK, a pigmented superficial lesion developed on the left cornea. The lesion migrated and recurred repeatedly and she was referred to our department. Best corrected vision was 20/220.

**Diagnoses:**

Light microscopy of a corneal scraping revealed branching fungal hyphae. Fungal culture showed growth of a black colony, identified as *E lecanii-corni* by ribosomal DNA sequencing.

**Interventions:**

We started topical treatment with 1% voriconazole and 1.5% levofloxacin. Antifungal susceptibility testing showed that the minimum inhibitory concentration of voriconazole was 0.06 μg/mL.

**Outcomes:**

The lesion scarred after treatment for 3 months, and left best corrected vision improved to 20/40.

**Lessons:**

Genus *Exophiala* is known as 1 of the “black molds” and a cause of chromomycosis. This is the first description of *E lecanii-corni* keratitis, and pigmented corneal epithelial lesions may be characteristic of this fungal genus.

## 1 Introduction

Patients with bullous keratopathy (BK) treated by Descemet stripping automated endothelial keratoplasty (DSAEK) have a compromised cornea, due to the administration of topical steroid, postsurgical use of contact lenses, and impaired barrier function of the corneal epithelium by BK. A compromised cornea risks infection by various opportunistic pathogens, including bacteria, yeasts and filamentous fungi.^[1]^ Among these, filamentous fungi include many rare pathogens not currently recognized as human pathogens. Here, we report a case of *Exophiala lecanii-corni* (*E lecanii-corni)* keratitis presenting as a serpiginous pigmented superficial lesion after DSAEK.

## 2 Case report

**Figure 1:**
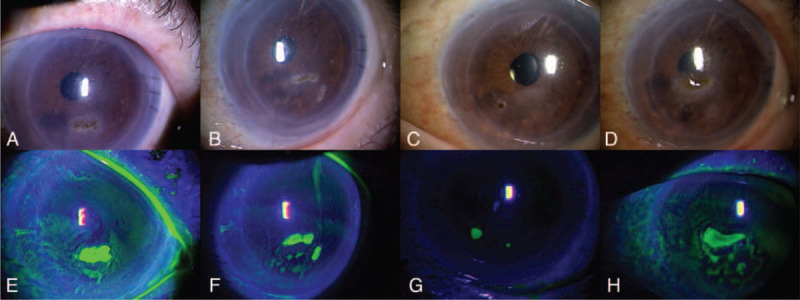
Photographs of corneal findings (A-D) and fluorescence staining (E-H) of the left eye taken by the previous doctor. Superficial corneal infiltrate with black pigmentation is observed by the previous doctor at time of onset (A, E). Treatment was started with corneal epithelial scraping and topical 1% natamycin ointment. The lesion migrated and recurred repeatedly: 1 month later (B, F), 4 months later (C, G), and 6 months later (D, H).

An 81-year-old woman who had undergone cataract surgeries at 50 years old developed decreased vision in the left eye. She had a history of hypertension, diabetes and unilateral nephrectomy for kidney tuberculosis. She visited a local doctor and was diagnosed with BK in both eyes. She then underwent DSAEK of the left eye and received postoperative follow-up with topical steroid and postsurgical use of a contact lens. Two months after DSAEK, a pigmented superficial lesion developed on the left cornea. Fungal keratitis was suspected and topical steroid was therefore tapered. Although corneal epithelial scraping and topical administration of 1% natamycin were performed, the lesion repeatedly migrated and recurred in other areas (Fig. 1). Nine months after the lesion first appeared, the patient was referred to our department. Topical steroid had been stopped for 1 month before first visiting our department.

**Figure 2:**
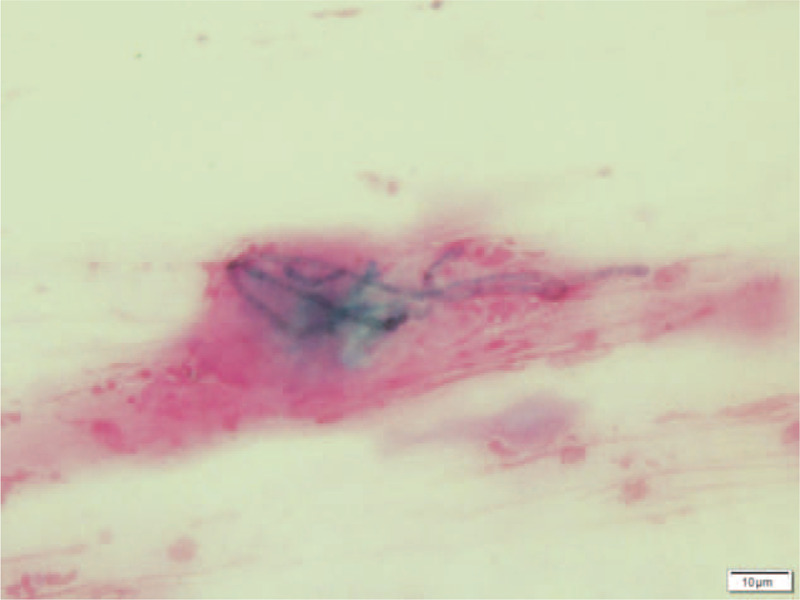
Smear of corneal scraping under light microscopy (Gram stain). Branching fungal hyphae are observed (×1000).

**Figure 3:**
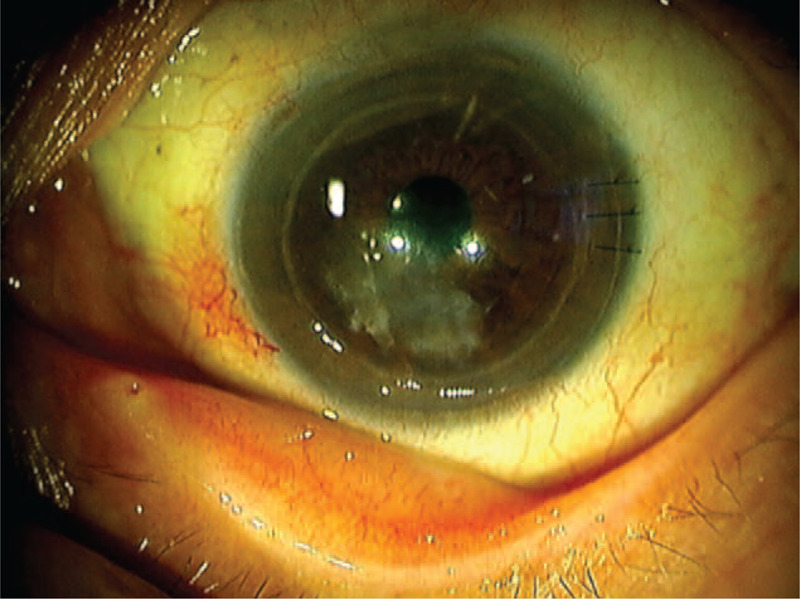
Slit-lamp examination shows scarred cornea 3 months after starting application of topical voriconazole. Best corrected visual acuity in the left eye was 20/40.

On the initial visit, best corrected visual acuity was 20/22 in the right eye and 20/220 in the left eye. Intraocular pressure was normal. Anterior segment examination of the left eye showed ciliary injection and superficial corneal infiltrate with an epithelial defect in a geographic shape, while the right eye appeared normal. Fundus examination yielded normal results for both eyes. Light microscopy of a Gram-stained corneal scraping revealed branching fungal hyphae (Fig. 2.). We therefore diagnosed fungal keratitis and started topical treatment with 1% voriconazole (VRCZ) hourly and 1.5% levofloxacin 3 times per day. Fungal culture showed growth of a colony with a velvety black surface. This isolated fungus was identified as *E lecanii-corni* based on sequencing of the internal transcribed spacer region of ribosomal DNA. Antifungal susceptibility testing was performed based on CLSI M38-A2. The minimum inhibitory concentration (MIC) for VRCZ was 0.06 μg/mL (Table 1) and topical VRCZ was continued. The lesion scarred after treatment with VRCZ for 3 months (Fig. 3). Left best corrected visual acuity at this point improved to 20/40 and no recurrence was observed.

**Table 1:**
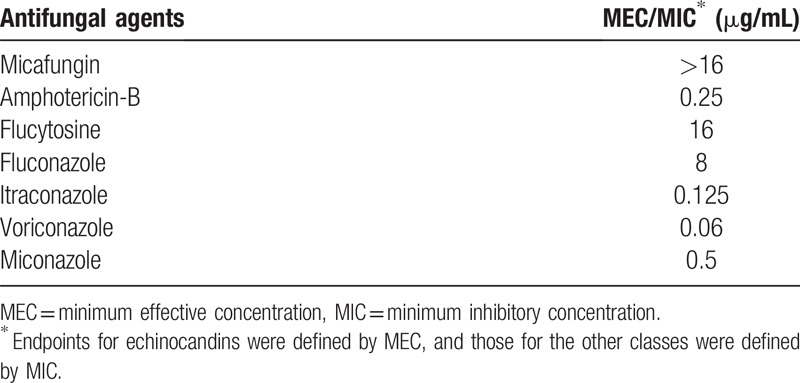
Susceptibility test of antifungal agents.

## 3 Discussion

Genus *Exophiala* is known as 1 of the black molds and a cause of chromomycosis.^[2]^ This genus is widely distributed in soil, plants and water sources. The fungus has also been isolated from living environments, such as dishwashers, steam bath facilities and bath rooms.^[3]^*Exophiala* species are morphologically variable and identification of the fungal species using sequence data of ribosomal RNA internal transcribed spacer regions is therefore recommended.^[2]^ Cases of *Exophiala* keratitis by *E dermatitidis*,^[4–6]^*E jeanselme*,^[7,8]^ and *E phaeomuriformis*^[9–11]^ have been reported following histories of trauma, corneal transplantation, laser in-situ keratomileusis, keratoprosthesis and long-term treatment with steroid. However, *E lecanii-corni* keratitis has not been reported, and this description represents the first report of keratitis caused by *E lecanii-corni*.

In most strains of *Exophiala* genus, in vitro antifungal susceptibility testing shows low MICs for amphotericin B, itraconazole and VRCZ.^[2]^ With the present strain, VRCZ showed a low MIC and the result was consistent with the observed clinical course for this case.

In our case, the corneal lesion had been confined to the surface for 1 year until treated. This case also presented with a specific clinical course in which the lesion migrated and recurred repeatedly. Pigmented superficial corneal lesions have been reported in several cases of *Exophiala* keratitis.^[5,6,8–11]^ Some kinds of filamentous fungus can secrete pigment during development under conditions suitable for growth. In this case, using a bandage contact lens and topical steroids may have contributed to pigment production.

In conclusion, we have reported a case of keratitis caused by *E lecanii-corni* after DSAEK. Characteristic corneal findings of a pigmented superficial lesion that migrated and recurred repeatedly were described. Molecular diagnosis was helpful in identifying this rare fungus, and treatment with topical VRCZ proved effective.

## Author contributions

**Conceptualizationt:** Tomoko Miyakubo, Daisuke Todokoro.

**Writing – original draft:** Tomoko Miyakubo, Daisuke Todokoro.

**Writing – review and editing:** Tomoko Miyakubo, Daisuke Todokoro, Yoshiyuki Satake, Koichi Makimura, Sumiko Miyakubo, Hideo Akiyama.
